# Design and Simulation of Logic-In-Memory Inverter Based on a Silicon Nanowire Feedback Field-Effect Transistor

**DOI:** 10.3390/mi13040590

**Published:** 2022-04-09

**Authors:** Eunwoo Baek, Jaemin Son, Kyoungah Cho, Sangsig Kim

**Affiliations:** 1 Department of Semiconductor Systems Engineering, Korea University, 145 Anam-ro, Seoul 02841, Korea; backeunwoo@korea.ac.kr; 2 Department of Electrical Engineering, Korea University, 145 Anam-ro, Seoul 02841, Korea; jaeminson@korea.ac.kr (J.S.); chochem@korea.ac.kr (K.C.)

**Keywords:** feedback field-effect transistor, logic-in-memory, mixed-mode simulation, positive feedback loop, silicon nanowire

## Abstract

In this paper, we propose a logic-in-memory (LIM) inverter comprising a silicon nanowire (SiNW) n-channel feedback field-effect transistor (n-FBFET) and a SiNW p-channel metal oxide semiconductor field-effect transistor (p-MOSFET). The hybrid logic and memory operations of the LIM inverter were investigated by mixed-mode technology computer-aided design simulations. Our LIM inverter exhibited a high voltage gain of 296.8 (*V*/*V*) when transitioning from logic ‘1’ to ‘0’ and 7.9 (*V*/*V*) when transitioning from logic ‘0’ to ‘1’, while holding calculated logic at zero input voltage. The energy band diagrams of the n-FBFET structure demonstrated that the holding operation of the inverter was implemented by controlling the positive feedback loop. Moreover, the output logic can remain constant without any supply voltage, resulting in zero static power consumption.

## 1. Introduction

Although the von Neumann architecture, a revolutionary development in the semiconductor industry, has improved integration density and performance in modern computers, physical separation between the processor and memory hierarchy causes energy-hungry data transfer and long latencies [1,2,3]. Considering the rise of data-intensive applications, such as artificial intelligence, the 5G communication standard, and Internet of Things since the fourth industrial revolution, a novel computing paradigm is essential for the massive data processing requirements.

The logic-in-memory (LIM) architecture is gaining attention owing to its space-saving structure and increased energy efficiency on integrating logic processes and data storage [4]. Most studies on LIM utilize emerging memories, such as resistive random-access memory (ReRAM) [5,6], spin-transfer torque RAM (STT-RAM) [7,8], and ferroelectric field-effect transistors (FEFETs) [9,10]. However, they comprise non-silicon components that are expensive and require additional fabrication procedures. Moreover, owing to the high off-current, ReRAM and STT-RAM require high supply voltages and peripheral circuits to guarantee a sufficient sensing margin [11,12]. Additionally, although FEFETs exhibit a relatively high ON/OFF current ratio, reducing the gate voltage based on the high voltage drop across the interface oxide is a challenge [13], one that limits the possibility of achieving high endurance. Therefore, LIM architecture comprising silicon-based devices needs to be explored further to utilize the metal-oxide-semiconductor (CMOS) technology while maintaining a simple structure and high endurance.

Therefore, in this study, we propose a CMOS-compatible LIM inverter comprising an n-channel feedback field-effect transistor (n-FBFET) made of a silicon nanowire (SiNW) and a SiNW p-channel metal-oxide-semiconductor field-effect transistor (p-MOSFET) made of a SiNW. FBFETs have demonstrated steep switching characteristics and gate-controlled memory behavior, making them a suitable choice for the LIM inverter [14,15,16]. Also, the stable performance of FBFET has been proved against charge trap and electrical bias stresses in recent research [17,18]. The proposed LIM inverter provides a high voltage gain while retaining the output logic at zero input voltage. Its memory behavior under zero supply voltage is a result of the FBFET storing electrons and holes in the channel region. Additionally, we demonstrated the hybrid logic and memory functions of the inverter using mixed-mode technology computer-aided design (TCAD) simulation, indicating the possibility of a novel computing paradigm beyond von Neumann’s computing.

## 2. Materials and Methods

All simulations were carried out using 2D structures via a mixed-mode simulation supported by the Sentaurus TCAD simulator (Synopsys Sentaurus (O_2018.06)), which is a commercial device simulator [19]. The physics models of n-FBFET and p-MOSFET include the Fermi–Dirac statistics, Auger recombination, bandgap narrowing, and Shockley–Read–Hall recombination with doping dependency, whereas the mobility models include doping dependence, Lombardi mobility, and high field saturation to analyze the electrical characteristics in the silicon region. We used the default parameters supported by Sentaurus TCAD simulator for all of the presented models. Additionally, surface Shockley–Read–Hall recombination was applied to the interface between silicon and Al_2_O_3_ in n-FBFET. In this study, all the simulations were performed for n-FBFET and p-MOSFET at 300 K.

## 3. Device Structure and Simulation

**Figure 1:**
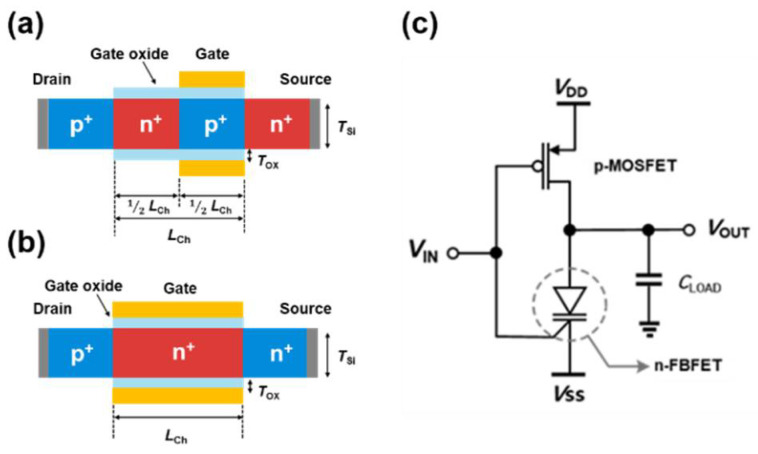
Cross section of a (**a**) SiNW n-FBFET and (**b**) a SiNW p-MOSFET. (**c**) Logic-in-memory (LIM) inverter comprising n-FBFET and p-MOSFET with load capacitor (*C*_LOAD_). Supply voltages *V*_DD_ and *V*_SS_ connected to the p-MOSFET and n-FBFET sources, respectively.

The cross-sectional views of an n-FBFET with a p^+^-n^+^-p^+^-n^+^ SiNW and a p-MOSFET with a p^+^-n^+^-p^+^ SiNW, and the circuit diagram of the LIM inverter are illustrated in Figure 1. The n-FBFET had dimensional parameters of a channel thickness (*T*_Si_) of 10 nm, a channel length (*L*_CH_) of 40 nm, and an Al_2_O_3_ gate oxide thickness (*T*_OX_) of 2 nm (Figure 1a). The channel consisted of the p^+^-doped region below the gate metal and the n^+^-doped non-gated region; each region had an identical length of 20 nm (1/2 *L*_CH_). The doping concentrations of the source, drain, and non-gated channel regions were 1 × 10^20^ cm^−3^. The gated-channel region was heavily doped with a p-dopant concentration of 7 × 10^19^ cm^−3^. For the p-MOSFET, *T*_Si_, *L*_CH_, and *T*_OX_ were 10, 40, and 2 nm, respectively (Figure 1b). The p-channel had a doping concentration of 1 × 10^19^ cm^−3^ and the doping concentrations of the source and drain regions were 1 × 10^20^ cm^−3^. The gate metal work functions were tuned with 5.65 eV for n-FBFET and 4.8 eV for p-MOSFET to obtain the optimal function in logic and memory operation. For the experimental implementation of the LIM inverter, Pt and heavily doped Si can be chosen as the gate metals for n-FBFET and p-MOSFET, respectively. The simulations were performed in the 2D structure via Synopsys Sentaurus [19].

The LIM inverter is based on a conventional CMOS inverter, comprising the n-FBFET as a replacement to n-channel MOSFET (n-MOSFET) and a p-MOSFET (Figure 1c). A load capacitor (*C*_LOAD_) of 1 fF was connected to the output node, assuming a parasitic capacitance existed between the line and logic gates. The circuit was biased with supply voltages *V*_DD_ and *V*_SS_ corresponding to the source voltages of p-MOSFET and n-FBFET, respectively, to calculate the output logic states, which were determined by sensing drain voltage of the n-FBFET (*V*_OUT_). The n-FBFET in the proposed inverter performs a key function in logic operation and data storage by implementing the memory function while retaining the conventional CMOS logic scheme structure. Moreover, the LIM inverter is fully compatible with the conventional CMOS process because silicon is used as the channel material. The LIM system based on our LIM inverter can be implemented experimentally by utilizing the conventional CMOS process and circuit.

## 4. Characteristics of the Proposed LIM Inverter

**Figure 2:**
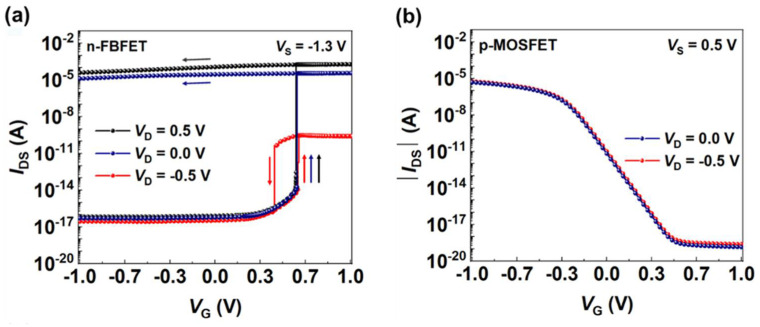
Simulated *I*_DS_–*V*_G_ transfer characteristics of (**a**) an SiNW n-FBFET with the drain voltages (*V*_D_) of 0.5, 0.0, and −0.5 V under a source voltage (*V*_S_) of −1.3 V and (**b**) a SiNW p-MOSFET with the *V*_D_ of 0.5 and 0.0 V under a *V*_S_ of 0.5 V.

Figure 2 show the transfer curves of the n-FBFET and p-MOSFET under several voltage conditions. The n-FBFET gate voltage (*V*_G_) ranges from −1.0 to 1.0 to −1.0 V to verify the hysteresis characteristics at *V*_D_ = 0.5, 0.0, and −0.5 V (Figure 2a). The latch-up phenomenon occurs during the forward sweep of *V*_G_, that is, *I*_DS_ increases steeply at *V*_G_ = ~0.6 V. The device shows an extremely low subthreshold swing (SS) of 2.3 × 10^−3^ mV/dec at *V*_D_ = 0.5 V, which is caused by the generation of the positive feedback loop in the channel region. After the latch-up phenomenon, the device transitions to the ON state, showing a high ON/OFF current ratio of 10^12^. However, when *V*_G_ sweeps reversely, *I*_DS_ decreases at *V*_G_ in a manner dissimilar to the latch-up phenomenon and is referred to as the latch-down phenomenon, after which the device transitions to the OFF state. The gap in *V*_G_ where the latch-up/latch-down phenomena occur indicates the memory window wherein the FBFET maintains the ON and OFF states of the device before the phenomena occur again. The ON/OFF current ratio and memory window become larger on applying more bias to *V*_D_. However, *V*_G_ remains unaffected.

Figure 2b shows the absolute value of *I*_DS_ versus *V*_G_ for p-MOSFET. As *V*_G_ decreases, the absolute value of *I*_DS_ approaches the saturation region at *V*_G_ = −0.5 V. The p-MOSFET exhibits over 60 mV/dec of SS due to the operation mechanism of thermal injection [20]; nevertheless, the high current ON/OFF ratio of ~10^15^.

## 5. Switching and Memory Operations in the LIM Inverter

**Figure 3:**
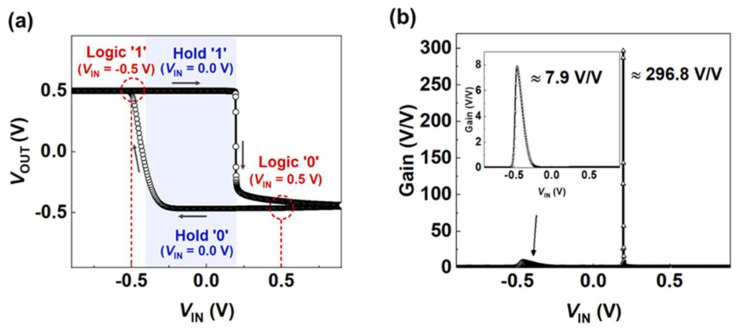
(**a**) Voltage transfer characteristics (VTCs) and (**b**) inverter gains of the LIM inverter under bias condition of *V*_DD_ = 0.5 V and *V*_SS_ = −1.3 V.

Figure 3a shows the voltage transfer characteristics (VTC) of the LIM inverter with supply voltages *V*_DD_ (0.5 V) and *V*_SS_ (−1.3 V). The output logic ‘0’ (or ‘1’) indicated the distinct low (or high) voltage value of *V*_OUT_ when an input voltage *V*_IN_ of 0.5 V (−0.5 V) was applied. Unlike a conventional CMOS logic inverter, the proposed inverter exhibits hysteresis characteristics, that is, the output logic states switch at different *V*_IN_. Therefore, the LIM inverter holds the logic data when *V*_IN_ = 0.0 V, as illustrated in Figure 3a. Hold ‘0’ and ‘1’ were determined by the processed logic state with *V*_IN_ = 0.0 V.

Figure 3b shows the inverter gains obtained from the absolute value of the differentiation of *V*_OUT_ from *V*_IN_. When p-MOSFET was turned on, the device transitioned from logic ‘0’ to ‘1’, and a relatively low inverter gain of ~7.9 *V*/*V* was observed, owing to the SS of over 60 mV/dec. Alternatively, logic ‘1’ steeply transitioning to ‘0’ resulted in a high gain of ~296.8 *V*/*V* owing to the latch-up phenomenon in n-FBFET. The LIM inverter operates in a narrow *V*_IN_ range because of the steep transition slopes. The *V*_IN_ range holding the logic data can be affected by temperature. Nevertheless, the LIM inverter still obtains a relatively sufficient voltage margin for memory operation since the device maintains the steep switching characteristics even under the temperature variation.

**Figure 4:**
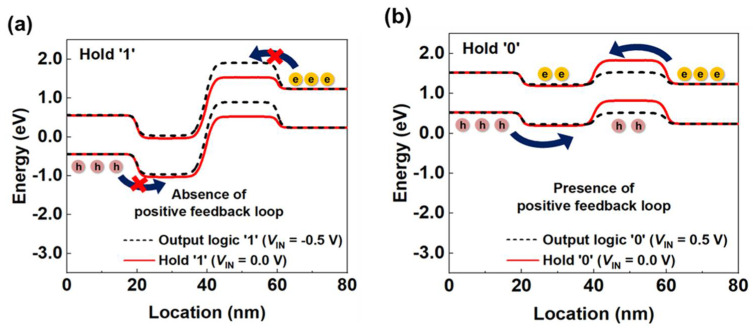
(**a**) Energy band diagrams of n-FBFET in the (**a**) output logic ‘1’ and hold ‘1’ and (**b**) output logic ‘0’ and hold ‘0’.

Figure 4 shows the conduction and valence bands of the n-FBFET to analyze the holding operation. The dashed lines and solid lines in red indicate the logic and hold states, respectively. When the output logic is ‘1’ (*V*_IN_ = −0.5 V), potential barriers were created in the channel region (Figure 4a), and the positive feedback loop is absent in the energy band diagram. The barrier height in the conduction band decreased as *V*_IN_ increased from −0.5 to 0.0 V. However, the potential barriers were high enough at *V*_IN_ = 0.0 V itself to block the injection of electrons into the channel region. Therefore, the energy level in the drain region remained constant, corresponding to hold ‘1’. Alternatively, when the output logic was ‘0’ (*V*_IN_ = 0.5 V), a positive feedback loop was seen in the conduction and valence bands (Figure 4b). As *V*_IN_ increases, the barrier height reduces and the electrons flow into the channel region and accumulate in the potential well, which causes a further decrease in the barrier height, and further induces injection of holes into the channel region. This iterative operation results in the collapse of the potential barrier, leading to activation of the positive feedback loop. As *V*_IN_ decreases from 0.5 to 0.0 V, logic ‘0’ is followed by hold ‘0’. Although the barrier height in the conduction band is higher, the charge carriers accumulated in the potential wells impede the regeneration of potential barriers, thereby enabling the device to maintain the energy level of the drain region that corresponds to hold ‘0’. Moreover, the FBFET is not affected by the tunneling mechanism during the operation. As for output logic ‘1’ and hold ‘1’, charge carriers are absent inside the intrinsic channel under the gate, and consequently the tunneling of charge carriers does not occur. On the other hand, for output logic ‘0’ and hold ‘0’, the tunneling of charge carriers cannot occur due to the flattened band structure after the positive feedback loop, even though charge carriers are present in the channel.

**Figure 5:**
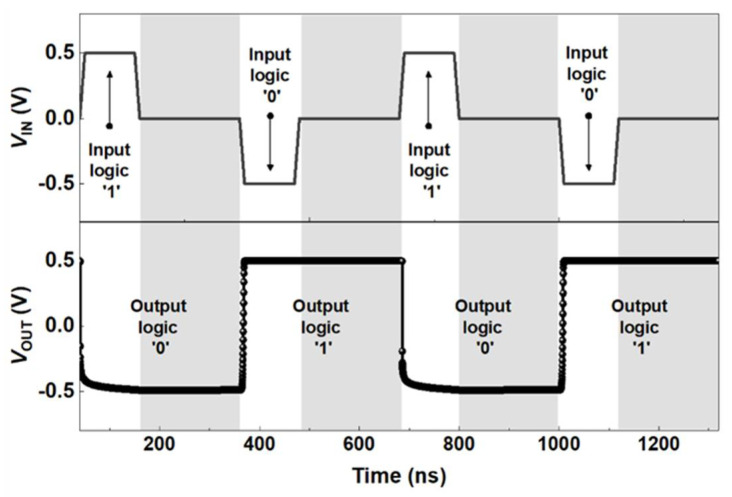
Timing diagrams of input and output voltages (*V*_IN_ and *V*_OUT_) with supply voltages *V*_DD_ = 0.5 V and *V*_SS_ = −1.3 V. *V*_IN_ is applied with a logic pulse width of 100 ns.

Further, the repetitive time response of the LIM inverter was verified by applying positive and negative input voltages with an absolute value of 0.5 V and a pulse width of 100 ns (Figure 5). To demonstrate the holding characteristics at *V*_IN_ = 0.0 V, *V*_IN_ is not pulsed for 200 ns after the logic process ends. The output logic transitions from ‘1’ to ‘0’, as a *V*_IN_ of 0.5 V is applied to input logic ‘1’. Conversely, the output logic switches to logic ‘1’, as a *V*_IN_ of −0.5 V is applied to input logic ‘0’. This stable logic process was conducted for 100 ns. It was observed that the inverter maintained a constant logic voltage value without voltage degradation, thereby verifying the logic processes and storage ability of the proposed inverter within a voltage range of −0.5 to 0.5 V for 100 ns, under the corresponding supply voltage conditions.

## 6. Operation of LIM Inverter under Zero-Bias Conditions

**Figure 6:**
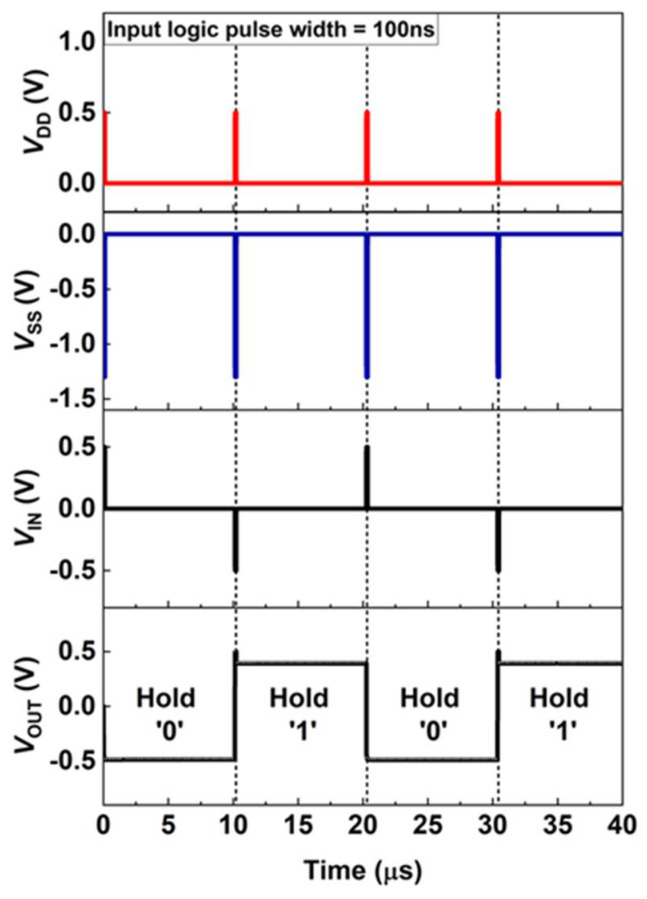
Holding characteristic with zero supply voltage. Timing diagrams of applied voltage (*V*_DD_, *V*_SS_ and *V*_IN_) with a logic pulse width of 100 ns and the corresponding output voltage (*V*_OUT_). The holding operation lasted for 10 μs.

Recently, FBFETs have demonstrated superior memory characteristics under zero-bias conditions by controlling the charge carriers accumulated in the channel region [16]. Thus, it was crucial to verify the memory behavior of logic circuits comprising FBFETs without supply voltages. As shown in Figure 6, the supply voltages *V*_DD_ and *V*_SS_ were input to the circuit with the same pulse width as that of the input logic pulse. Hold ‘0’ and ‘1’ (*V*_IN_ = *V*_DD_ = *V*_SS_ = 0.0 V) lasted for 10 μs after the output logic is processed. When input logic ‘0’ is applied for 100 ns with a *V*_DD_ of 0.5 V and a *V*_SS_ of −1.3 V, the LIM inverter displays the output logic as logic ‘1’. Further, when supply voltages were removed, *V*_OUT_ decreased slightly and was affected by the current through p-MOSFET. Nevertheless, *V*_OUT_ remained constant for hold ‘1’ because the potential barriers in n-FBFET prevented further injection of charge carriers. When input logic ‘1’ was applied with the same supply voltages, the output logic transitioned from logic ‘1’ to ‘0’. For hold ‘0’, *V*_OUT_ consistently retained the initial value as of output logic ‘0’ without any voltage drops. As the charge carriers were accumulated in the n-FBFET channel region, logic ‘0’ remained consistent by maintaining the positive feedback loop, which allowed the LIM inverter to retain data in the absence of a voltage supply. Furthermore, the LIM inverter did not consume static power because *V*_DD_ and *V*_SS_ became 0.0 V. Since the static power is calculated as a multiple of supply voltage and current through the circuit, the LIM inverter consumed zero static power during hold ‘0’ and ‘1’ while not requiring alternate peripheral circuits.

**Figure 7:**
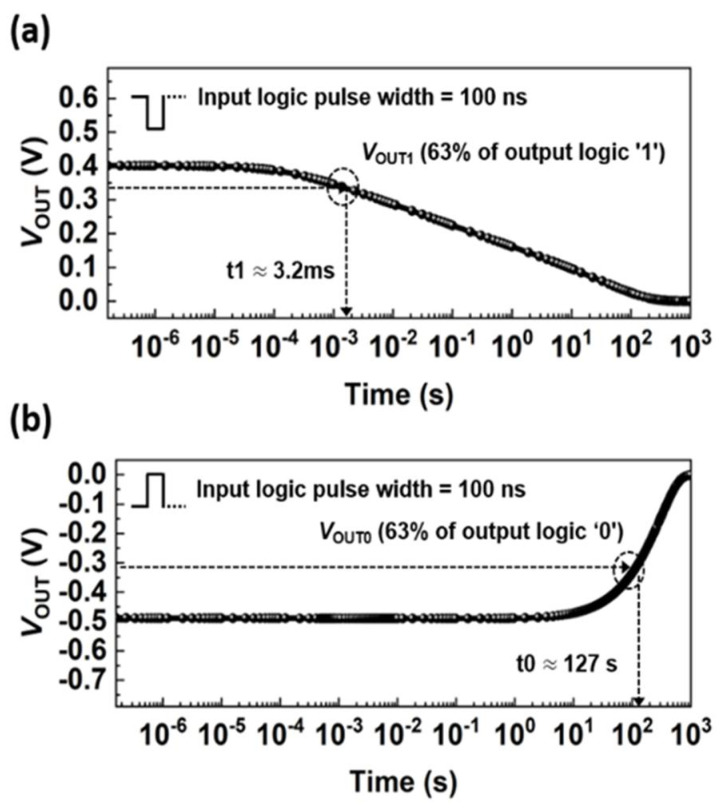
*V*_OUT_ versus time functions for holding the (**a**) output logic ‘1’ and (**b**) output logic ‘0’ under *V*_IN_ = *V*_DD_ = *V*_SS_ = 0.0 V. t0 and t1 indicate the time when *V*_OUT_ reaches 63% of initial voltage of output logic ‘0’ and ‘1’ during holding operation.

Figure 7 shows the *V*_OUT_ values of the time function after calculating the logic state for 100 ns to confirm the possible extent of the holding operation under *V*_DD_ = *V*_SS_ = *V*_IN_ = 0.0 V. As time was increased to 1000 s, *V*_OUT_ gradually approaches zero voltage during the holding operation, which affects the continuous leakage current running through the circuit. The time values when *V*_OUT_ increases to 63% of its initial value, were denoted as t0 and t1 for logic ‘0’ and ‘1’, respectively. At 63% of the initial logic ‘1’, t1 was 3.2 ms (Figure 7a). Alternatively, logic ‘0’ takes much longer to lose the stored logic ‘0’, and, hence, t0 was ~127 s (Figure 7b). It was worth noticing that logic ‘0’ showed a substantially long t0 over 100 s, based on the charge carriers accumulated in the n-FBFET channel region. As a result, the proposed inverter can store over 63% of output logic voltage in 127 s (3.2 ms) for logic ‘0’ (‘1’) without consuming static power.

## 7. Conclusions

We demonstrated the hybrid logic and memory operation of an LIM inverter using mixed-mode TCAD simulations. The inverter exhibited voltage gains of ~296.8 (*V/V*) when transitioning from logic ‘1’ to ‘0’ and 7.9 (*V*/*V*) when transitioning from logic ‘0’ to ‘1’, and it processed the output logic within 100 ns. The simulated energy band diagrams of n-FBFET demonstrated the holding operations implemented with zero input voltage by controlling the positive feedback loop. Furthermore, the proposed inverter was able to retain 63% of the initial output logic of logic ‘1’ and logic ‘0’ for up to 3.2 ms and 127 s, respectively, without supply voltages. The above results verify the possibility of merging logic and memory operations using the proposed LIM inverter while consuming zero static power.
